# “Positive Peers”: Function and Content Development of a Mobile App for Engaging and Retaining Young Adults in HIV Care

**DOI:** 10.2196/13495

**Published:** 2020-01-30

**Authors:** Mary M Step, Jennifer McMillen Smith, Joshua Kratz, Julia Briggs, Ann Avery

**Affiliations:** 1 College of Public Health Kent State University Kent, OH United States; 2 Metrohealth Medical Center Cleveland, OH United States; 3 Moffitt Cancer Center Tampa, FL United States; 4 Blue Star Design Cleveland, OH United States; 5 Case Western Reserve University School of Medicine Cleveland, OH United States

**Keywords:** HIV, young adults, mobile applications, self-management

## Abstract

**Background:**

Although treatment for HIV infection is widely available and well tolerated, less than 30% of adolescents and young adults living with HIV infection achieve stable viral suppression. Mobile technology affords increased opportunities for young people living with HIV to engage with information, health management tools, and social connections that can support adherence to treatment recommendations and medication. Although mobile apps are increasingly prevalent, few are informed by the target population.

**Objective:**

The objective of this study was to describe the “Positive Peers” app, a mobile app currently being evaluated in a public hospital in the Midwestern United States. Formative development, key development strategies, user recruitment, and lessons learned are discussed in this paper.

**Methods:**

“Positive Peers” was developed in collaboration with a community advisory board (CAB) comprising in-care young adults living with HIV and a multidisciplinary project team. Mobile app functions and features were developed over iterative collaborative sessions that were tailored to the CAB members. In turn, the CAB built rapport with the project team and revealed unique information that was used in app development.

**Results:**

The study was funded on September 1, 2015; approved by the MetroHealth Institutional Review Board on August 31, 2016; and implemented from October 11, 2016, to May 31, 2019. The “Positive Peers” mobile app study has enrolled 128 users who reflect priority disparity population subgroups. The app administrator had frequent contact with users across app administration and study-related activities. Key lessons learned from the study include changing privacy concerns, data tracking reliability, and user barriers. Intermediate and outcome variable evaluation is expected in October 2019.

**Conclusions:**

Successful development of the “Positive Peers” mobile app was supported by multidisciplinary expertise, an enthusiastic CAB, and a multifaceted, proactive administrator.

## Introduction

### Background

By the end of 2016, over 254,000 young people aged between 13 and 34 years were living with HIV in the United States [1,2]. Although young adults had similar care patterns as older patients living with HIV, they were less likely to have or adhere to antiretroviral therapy and achieve viral suppression [3-5]. Among those newly diagnosed, the most affected subpopulations are young, black, or Hispanic men who have sex with men [6]. Once engaged in HIV care, young adults also face social and economic barriers that jeopardize viral suppression. For example, younger adults are more likely to live in low-income households, to have been recently homeless or incarcerated, and to be uninsured or have only Ryan White Program–funded health care [7-10]. Not surprisingly, gay and bisexual teens and young adults report feelings of isolation and lack of support [3,11-13].

Young adults’ high behavioral risk profile is compounded by the stigma associated with HIV and generally low HIV literacy among this group [11,14]. Along these lines, a Kaiser Family Foundation survey showed that 51% of young adults aged between 18 and 30 years said that they would be uncomfortable having a roommate with HIV and 58% said that they would be uncomfortable having their food prepared by someone with HIV [15]. More than half of this sample agreed with the false belief that HIV can be transmitted by spitting or kissing. Perceived stigma and misperceptions about HIV may prevent young adults from disclosing their HIV status to others or seeking HIV care [16,17]. Given these challenges, there is a need to develop interventions that offer safe entry to long-term, continuous, and coordinated HIV care and address the varied socioeconomic and psychosocial needs of young people living with HIV (YPLWH) [18-22].

HIV research reflects a legacy of innovation and commitment to engaging patients in the care cascade. For example, boosting resilience, seek and test strategies, single-tablet treatment regimens, and same-day antiretroviral therapy availability have all been shown to attract hard-to-reach patients to treatment [23-26]. This spirit of innovation has recently been applied to newer modes of technology and communication that reach many more patients, particularly young people.

A growing number of mobile apps have been introduced, that offer users a range of functionality [27-31]. Mobile apps afford users with increased accessibility to clinicians, tools for self-management, and connect geographically dispersed members of a disease- or illness-defined group [30,32,33]. Over time, these networks can serve as a significant source of emotional support, a known protective factor during chronic illness [34-36].

Although mobile apps that support YPLWH are being implemented with greater frequency [31,37-42], most focus on medication adherence [43]. In addition, there are few available mobile apps offering a broad complement of functionality that includes both group- and interpersonal-level peer connections. Peer influence regarding engaging in risk behavior and decision making is strongest among adolescents and younger adults [44]. Within the context of social media networks, peer approval and peer feedback has been associated with improved self-esteem [45], learning [46], and perceived social support [47].

“Positive Peers” is a Web-based, mobile app that serves as a tool for young adults diagnosed with HIV to manage their health, establish connections with local resources, and engage in a supportive peer community (See Multimedia Appendix 1). Although this app is currently being evaluated in the field, increasing demand to create digital tools for key populations motivated us to share our formative approach and lessons learned [17,27]. This effort is part of the Health Resources and Services Administration (HRSA), HIV/AIDS Bureau’s (HAB’s) Special Projects of National Significance (SPNS) Program initiative, *Use of Social Media to Improve Engagement, Retention, and Health Outcomes along the HIV Care Continuum*. This is the first report of an ongoing study created to design, implement, and evaluate “Positive Peers.”

### Theoretical Foundation

“Positive Peers” is informed by media affordances theory, which specifies that communication qualities of technologies provide opportunities to act in a specific way relative to a user’s needs [48-51]. Although the features of a technology can influence a user’s perceptions of an app’s utility, these features do not determine user affordances alone. For example, a chat feature on a social media platform may be desirable to a potential user, but availability alone will not determine if or how often that user will actually use the feature. Affordance is not established until the user has a chance to explore and use the chat feature. Users’ perceptions of affordances emerge from interaction with the technology rather than from the technology itself [48,52].

The “Positive Peers” app is a social and technological behavioral *process* that affords the user opportunities to meet perceived needs, such as HIV-relevant information or supportive companionship. App functions reflect known affordances (eg, accessibility and social presence) and are intended to reflect qualities of social (ie, communication) transactions [48]. As we expect users’ needs to change across users and over time, the app is designed to provide a range of information and support services.

## Methods

### Formative Development Phase

“Positive Peers” was prompted by positive experiences with a clinic-sponsored private Facebook page shared by several young adults retained in HIV care. This group comprised 63 in-care patients who consistently attended support groups at the clinic. The age of this group ranged from 13 to 55 years, and 65% (41/63) of them were aged under 35 years. The group included 16 females and 47 males, and 75% (47/63) of them were people of color. As Facebook users thought that a mobile app would be far preferable, the HIV social worker who ran this group met with the users to identify desirable characteristics and potential functions of a HIV-centric mobile app. These specifically included chat, reminder, and resource functions. Consequently, the social worker explored the idea of a locally based mobile app (now named “Positive Peers”) with a hospital physician specializing in HIV.

### Multidisciplinary Project Team

The key members of the “Positive Peers” project team include the clinicians described above; a social scientist with a PhD and training in health communication theory and intervention development for evaluation; and a trained biostatistician to manage, link, and evaluate app data. A local design firm with health care marketing experience was invited to manage the creative activities of the project. The chief executive officer (CEO), a trained graphic designer and creative writer, is the creative director of the project and oversees the development of visual and written material. Finally, a local digital development firm, with significant experience building and maintaining health-related websites and apps, was brought onto the project to create the app architecture from the ground up. The director of app development and the senior developer are both trained in computer science, programming, and coding. They are also responsible for creating tracking tools to reliably record all app activity while it is in the field. The project administrator, selected post funding award, is a community HIV activist trained in public health administration. All activities that followed were conducted under the auspices of the MetroHealth Hospital’s Institutional Review Board.

### Community Advisory Board

The formative work for “Positive Peers” continued with the creation of a community advisory board (CAB). As user collaboration is key to the development of population-specific health technology [53-56], we asked this group to generate ideas that defined the tone, look, and functionality of the “Positive Peers” mobile app. The group meets with the development and implementation team on an ad hoc basis to address app-related concerns. They are compensated for their time and provided a catered meal. The CAB group demographics are 78% (29/37) male, 9% (3/37) transwomen, 81% (30/37) black or African American, 86% (32/37) aged less than 30 years, and 84% (31/37) who identify as Lesbian, Gay, Bisexual, Trans, or Queer (LGBTQ. Although the CAB group is large (n=37), not all members are present for every meeting, which tends to include 12 to 15 members.

To maintain a lively and productive atmosphere, a number of structured techniques were used to collect feedback in the CAB meetings, including card sorting, brainstorming, dyadic and small group idea generation, and traditional focus group methods. The CAB members are sometimes presented with booklets containing illustrated options or screenshots for app functions and features. The CAB first met with team members to view and vote on color schemes and avatar styles and discuss what functions might be most important to them in an HIV-centric app. Their primary concern at this meeting was privacy. They did not want anyone looking over their shoulder to see *HIV* in the name or function titles and preferred cartoon avatars and made up usernames to selfies and actual names. They also collaboratively imagined app features, including personal stories, a community forum, a resource page, medication, and appointment reminders.

### “Positive Peers” Prototype

Following the translation of the CAB’s initial ideas by graphic design and digital team members, a prototype (ie, alpha) of the “Positive Peers” app was presented at a joint CAB and project staff meeting. The digital engineers helped the CAB members download and navigate the prototype functions, whereas the design and marketing organization gauged the reaction to the app’s appearance, titling, content, and other design elements. This first meeting provided an opportunity for the nonclinical staff to get to know the CAB members and consider ways to further refine messaging and design. The project staff observed the reactions of the CAB members and noted questions and comments. In general, the CAB members expressed enthusiasm seeing their ideas come to life, which created increased anticipation for a beta version of the app. Subsequent team debriefing concluded that users found the app easy to download and that the app functions were intuitive and potentially useful. Although the CAB recommended against easily identifiable usernames, there were some concerns about users requiring frequent username and password reset.

A second CAB event was held a few months later to gain insight on the preferred messaging and content strategies. To attract as many CAB members as possible, this interactive CAB event took on a carnival theme that featured incentivized games designed to collect the CAB members’ needs, perceptions, and concerns about living with HIV as well as inform the tone and appearance of the app. For example, in exchange for a chance to play a game (eg, ping pong ball toss and Plinko), participants needed to offer a blog topic suggestion, a stigma-inducing or stigma-reducing suggestion, or name a function (eg, *Tales of Triumph*), depending on the purpose of that station. In a more private setting, personal stories of living with HIV were recorded by an interviewer, whereas a professional photographer took artistic portraits of the storyteller. Audio and photo consent were collected before this activity. Participants were also given the option to have a photograph that hid their facial features if they wished to maintain anonymity. The team designed colorful game booths and provided inexpensive carnival-type prizes, cotton candy, soda pop, and hot dogs to match the theme.

These early CAB meetings informed the primary app functions and directed the design team toward topics, issues, and images desired by potential users. As illustrated in Table 1, the social media affordances scale was used to map the desired functions to empirically derived affordances of various social media. We aimed to ensure broad coverage of these affordances to maximize usability among our population. Future evaluation will assess perceived affordances and investigate their association with study outcomes.

**Table 1:** “Positive Peers” functions mapped to social media affordances.

App function	Affordance	Affordance definition
Reminders, blogs, daily inspiration, forum, chat, and entire app	Accessibility	Convenient to use, easy to message anytime, and few structural barriers or gatekeepers
Blogs, daily inspiration, forum, and chat	Bandwidth	Richness of information one can convey (eg, nonverbal cues and expressed emotion)
Forum, chat, daily inspiration, and tales of triumph	Social presence	Perceived interpersonal closeness or immediacy of others on the channel
Chat	Privacy	Only the user can see the communication or messaging with another
Forum	Network association	Multiple users can participate in an interaction and other users can see who is communicating with whom
Reminders, forum, and chat	Personalization	Freedom to tailor messages and direct communication to select people
Blogs, tales of triumph, forum, and resources	Persistence	Permanence of messaging, permanence of content, and a record of activity
Reminders, forum, and chat	Editability	Ability to delete or edit messaging and ability to edit before posting
Forum and chat	Conversation control	Freedom to enter or end conversations
Forum, chat, and entire app	Anonymity	Channel blinds the user’s identity or allows the user to create an identity

At this stage of development, the development team needed to carefully consider the app size. Although we had the plan and expertise to include a wide range of features, we could not deliver an app that was so big that it was quickly deleted by users with budget smartphones. A review of existing popular apps suggested that “Positive Peers” must not exceed 150 MB. For example, popular apps such as *Facebook* and *Snapchat* can range from 400 MB to 650 MB, whereas *Instagram* (200 MB), *Yelp* (150 MB), *Twitter* (110 MB), and some weather apps (120 MB) are much smaller. We aimed to deliver an app comparable in size with those on the lower end. Currently, *“Positive Peers”* app requires 117 MB of smartphone memory to use. Our next step was to translate our CAB recommendations into a testable digital app with targeted content.

### “Positive Peers” Content Development

“Positive Peers” serves as both an information hub for newly diagnosed participants and a recruiting tool for eligible out-of-care community members. Therefore, a broad scope of content is available within the app and via a network of “Positive Peers” branded social media platforms, including *Facebook*, *Instagram*, and *Twitter*. Select “Positive Peers” content is also available on the project website [57], although most features are limited to authorized users on the mobile app. The purpose of related social media content is to disseminate accurate HIV education to young people, while also creating positive brand perceptions of “Positive Peers” within the larger HIV community.

“Positive Peers” blog content is researched, written, and presented by the design team. Before content development, a social media posting protocol was developed by the project team in collaboration with MetroHealth. “Positive Peers” text and imagery (eg, blog or social media posts and inspirational stories) are designed to maintain a consistent voice that is proactive, energetic, affirming, contemporary, and accurate. The CAB members asked for information to be realistic and open about the challenges of sex, dating, gender orientation, and LGBTQ issues. Messaging aims toward projecting inclusivity and tolerance while also maintaining a sense of humor and care for people who may follow our messaging. As recommended by the US Department of Health and Human Services, all content is designed not to exceed a seventh-grade reading requirement [58] and is vetted by both the project team and the CAB members. CAB meetings revealed that our population prefers edgier content that includes sexually graphic images, photos of real people, and humorous approaches. They requested diversity of gender, race, and fashion style in all photographic and illustrated artwork.

The app development was time and labor intensive. Progress was affected by several unpredictable factors, including CAB availability, organizational constraints (eg, legal negotiations), and funder requirements. The app development process benefited from allowing time for key team members to become familiar with each other’s work process, vocabulary, and personalities. The project manager implemented project organization (eg, meeting agenda and action plans), monitored frequent in-person meetings, and utilized project management tools for electronic to-do lists, group discussion, and document archives. “Positive Peers”*’* development phase took 1 year to develop the prototype and an additional 4 months to deliver a beta version.

### The “Positive Peers” App

The “Positive Peers” app comprises 3 general functions: (1) health management, (2) resources, and (3) social networking. The app affords several features that support these functions.

#### App Function 1: Health Management

The “Positive Peers” app offers 3 tools for managing HIV-related health needs. These include a reminder tool, lab tracker, and daily inspirational support. Each of these health management tools are described briefly below.

#### Reminders

**Figure 1:**
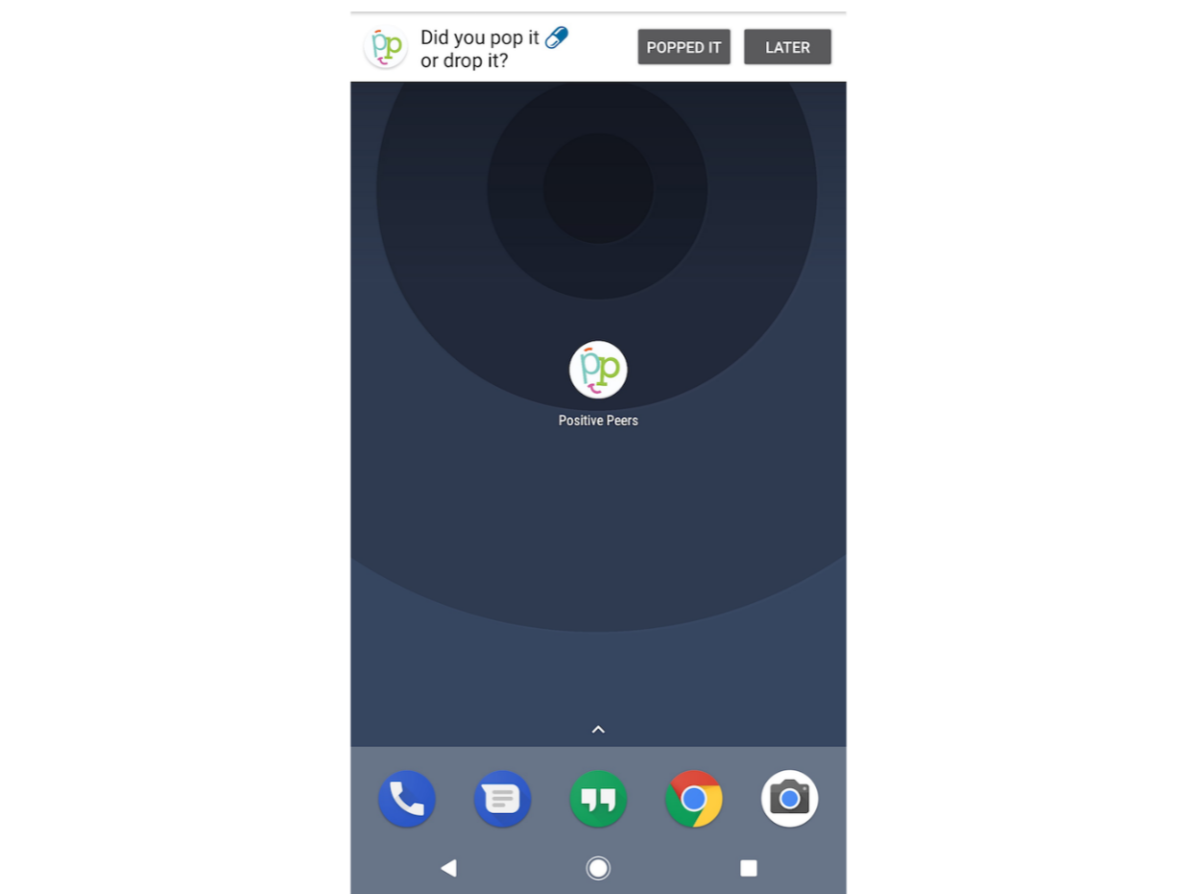
Screenshot of the interactive medication reminder.

This feature allows users to create an appointment or medication reminder that is received directly via push notification on their mobile device. Upon receipt, the user can confirm, snooze, or ignore the reminder directly from the received push notification (Figure 1). As push notifications can appear suddenly on a locked home screen, the language is brief, informal, and does not mention HIV specifically (eg, “Did you pop it or drop it?”). This is to protect the user’s privacy, while also making the notification easy for the user to recognize and understand. The reminder function also offers users an opportunity for tailoring this function by selecting to opt out of various push notifications.

#### Lab Tracking

CD4 counts and viral load values are the primary indicators of HIV disease progression and response to therapy. “Positive Peers” users have the ability to manually input their CD4 count and viral load values after they receive a lab report. This function is also optional so that the users can choose whether or not to store protected health information in the app.

#### Daily Inspiration

As a means of supporting emotional well-being, the app uses push notifications to deliver daily inspirational messages from the project administrator. These simple, affirming messages (eg, “If it feels like nothing is going right, go left.”) are sent to all registered app users. Brief inspirational messages are drafted by the design team, vetted by the clinical staff, and released on weekdays. These are aimed to (1) normalize fears and uncertainties; (2) create a humorous, positive, and action-oriented tone; and (3) reinforce reaching out for support.

#### App Function 2: Resources

The *Resources* tab on the “Positive Peers” app offers 3 features: (1) *Tales of Triumph*, a collection of CAB-created personal stories; (2) *The Blog,* a blog feed of culturally affirming content; and (3) *Local Resources*, a curated list of local organizations and services that may be of interest to young adults living with HIV.

**Figure 2:**
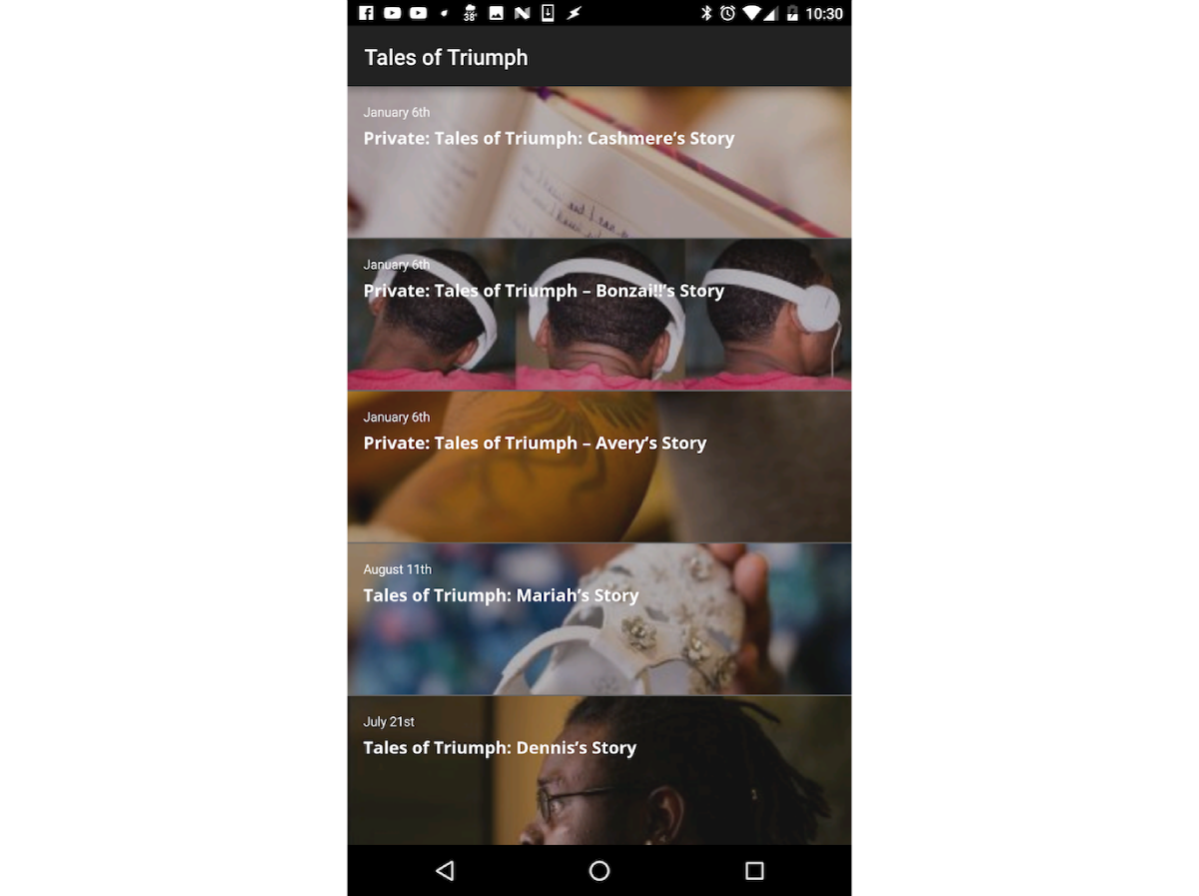
Screenshot of the Tales of Triumph homescreen.

**Figure 3:**
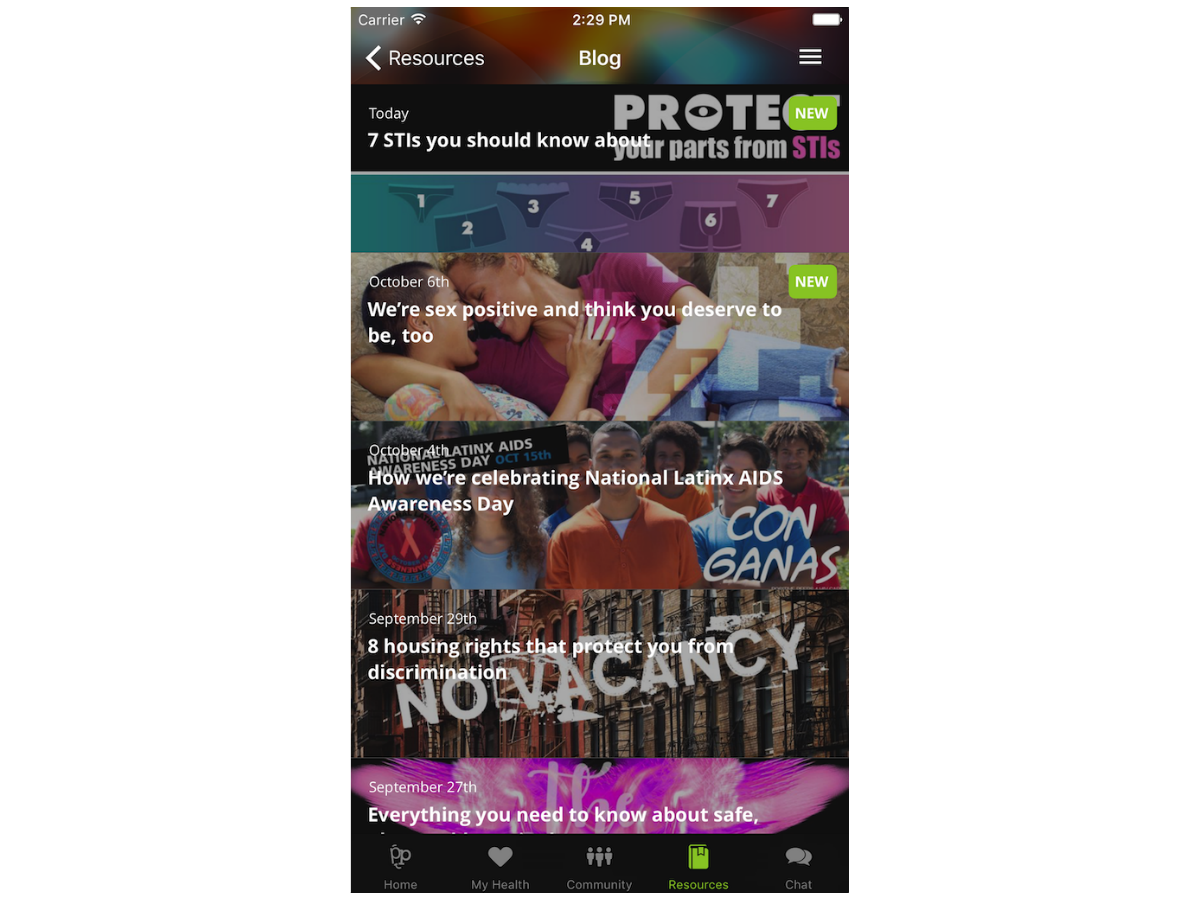
Screenshot of The Blog.

*Tales of Triumph* is a collection of brief, first-person narratives of personal challenges written by several community members of “Positive Peers” (Figure 2). These voluntarily provided stories are intended to be an inspirational and motivating opportunity for new users to identify with the community. Stories often reflect personal journeys through diagnosis, status disclosure, and other stigma-related challenges, emphasizing solutions and lessons learned. The Blog is a dynamic stream of informative, HIV-centric topics that appear on the Resources tab of the app as well as on the publicly available “Positive Peers” website and social media posts. Many entries reflect HIV health and wellness education, various community resources, and lifestyle features (Figure 3). Topics are categorized in an index for searchability. Past topics include “I’m worried I was exposed to HIV,” “What happens if I don’t seek treatment for HIV?,” and “Reasons you may feel tired.” The Blog also provides a recurring video blog with the project administrator (Hey Josh!) and the HIV social worker on the project (What’s up Jen?). These posts feature people known and liked by the “Positive Peers” community and typically generate several user comments.

The *Resources* tab provides a selection of current local community resources such as housing assistance, support groups, food and clothing sources, and substance abuse programs. There is also a directory of the hospital HIV clinical staff using which a patient can call or email their contact information with the tap of their finger.

#### App Function 3: Peer Networking

An HIV diagnosis is a life-changing, stressful experience compounded by social stigma and loss of support. However, social connection is a vital resource known to predict physical and mental health and promote resilience in HIV care [35,59]. “Positive Peers” was designed to increase social connectedness for YPLWH via the following 2 functions: community forum and chat.

#### Community Forum

**Figure 4:**
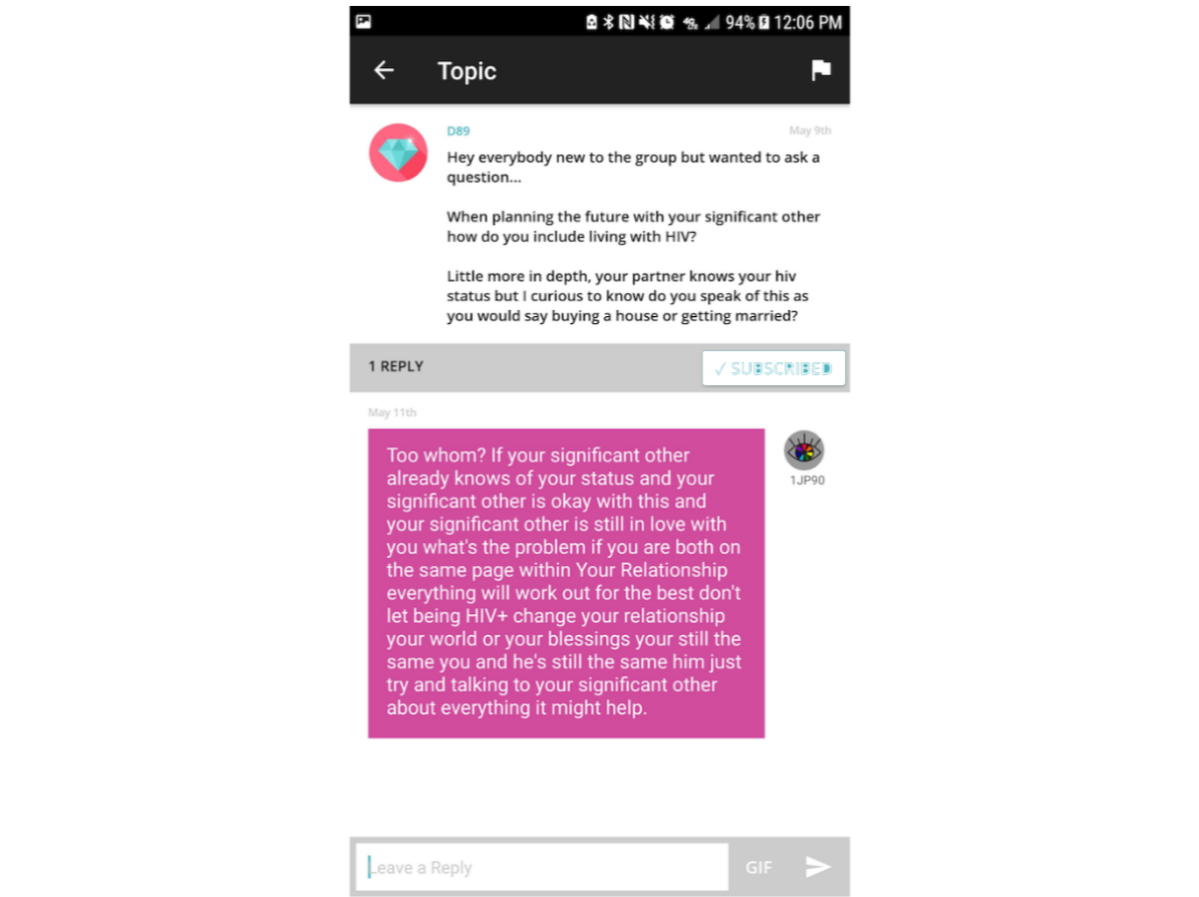
Screenshot of the Community Forum.

“Positive Peers” offers 2 distinct social networking functions. The first is a virtual community for registered users moderated by the project administrator. This app feature is intended to help users overcome feelings of isolation and to facilitate supportive and informational conversations (Figure 4).

Users are able to post their own discussion threads or respond to those posted by other users. Before gaining access to the app, all users received a copy of rules regarding civility and barring sexual provocation. Users are then required to sign a form indicating formal acknowledgment of the rules and agreement to abide by the protocol. As the CAB expressed a strong need for safety in the community forum, this feature offers a flag, which when clicked will allow a user to report any misbehavior directly to the administrator.

#### Chat

The chat feature allows users to message another user or the administrator privately. Chat affords users the ability to connect with one another and share personal information (eg, phone number and real name) at their discretion. Chat also provides the project administrator a quick and reliable way to communicate directly with any app user. Importantly, the chat function is stratified by age group. Minor users (<18 years) are segregated from adults to protect them from inappropriate posts or solicitations. The administrator retains the ability to communicate with everyone.

### Project Administrator

The project administrator is a central figure to the “Positive Peers” network and important to the daily functioning of the app. The person in this role manages recruiting, referrals, and onboarding procedures; monitors app-based interaction; responds to users’ questions and password reset requests; coordinates evaluation activities; and generally, serves as the human face of “Positive Peers” in the clinic and the community. This role requires significant flexibility, problem-solving ability, and social skill. Our “Positive Peers” population is generally highly transient and cautious of others. Our administrator is perceived as a group insider and advocate.

Updates and bug fixes are a natural part of sustaining a mobile app in the marketplace. Although the app is closely monitored, bugs have been reported by users to the administrator, who works closely with the app design specialist to pinpoint and address the issue. Both planned and unplanned updates are part of regular operating conditions. Planned updates typically reflect expansion, new features, or platform updates. Unplanned updates tend to address bugs, unanticipated platform updates, and other issues that threaten normal operations.

## Results

### Participants

“Positive Peers” is designed for use by YPLWH. The design and content of the mobile app are created to be relevant to young people of low socioeconomic status and of racial/minority backgrounds. Although “Positive Peers” was developed for adolescents and young adults, the app could be modified to target any particular user group. The eligibility requirements for “Positive Peers” included (1) being aged between 13 and 34 years, (2) ability to speak English, (3) having a clinical diagnosis of HIV, (4) having either a new diagnosis (within the past year) or being out-of-care for 6 or more months (within the last 2 years) or not virally suppressed (>200 viral load), and (5) willingness to receive care at the MetroHealth HIV clinic.

Table 2 illustrates the participant characteristics to date. Only 2 adolescent (<18 years) participants are enrolled in the app, and the majority of users (100/128, 78.1%) reported their ages as between 18 and 30 years. Additional descriptors showed that this user sample was primarily non-Latinx (113/128, 88.3%) and African American (87/128, 68.0%). People who reported LGBQ+ orientation comprised 75.8% (97/128) of the group, and a small set of users identified their gender identity as something other than cisgender (7/128, 5.5%). Although almost 20.3% (26/128) of users did not graduate high school (or attain a General Education Diploma), most have experienced some college (59/128, 46.1%). One-third of the users were diagnosed within 1 year of enrollment. Generally, these users were aged under 30 years and were LGBTQ people of color, who had been living with HIV for 5 years or less.

**Table 2:** “Positive Peers” users’ demographic characteristics (n=128).

Characteristics		Value, n (%)
**Age (years)**		
	13-17	2 (1.6)
	18-24	42 (32.8)
	25-29	58 (45.3)
	30+	26 (20.3)
**Race**		
	White	25 (19.5)
	African American	87 (68.0)
	Multiracial or other	16 (12.5)
**Latinx**		
	No	113 (88.3)
	Yes	15 (11.7)
**Sexual orientation**		
	Heterosexual	31 (24.2)
	Lesbian or gay	60 (46.9)
	Bisexual	27 (21.1)
	Queer	3 (2.3)
	Other	7 (5.5)
**Gender identity**		
	Cisgender male	101 (78.9)
	Cisgender female	20 (15.6)
	Trans/transgender man	1 (0.8)
	Trans/transgender woman	4 (3.1)
	Genderqueer or gender nonconforming	1 (0.8)
	Other	1 (0.8)
**Education**		
	Not a high school graduate	25 (19.5)
	High school graduate and no college	44 (34.4)
	Some college	59 (46.1)
**Employment status**		
	Full-time employment	37 (28.9)
	Part-time employment	28 (21.9)
	Unemployed	51 (39.8)
	Student	6 (4.7)
	Disabled	6 (4.7)
**Incarceration**		
	Never	63 (49.2)
	1-2 times	34 (26.6)
	3-5 times	23 (18.0)
	>5 times	8 (6.3)
**Born with HIV**		
	No	116 (90.6)
	Yes	12 (9.4)
**First diagnosed with HIV**		
	Within the past 12 months	42 (32.8)
	More than 12 months ago	86 (67.2)
**Living with HIV**		
	1 year or less	47 (36.7)
	2-5 years	38 (29.7)
	6-10 years	23 (18.0)
	>10 years	20 (15.6)

### Recruitment and Retention

#### Recruitment

The most effective form of recruitment for “Positive Peers” was in-person interactions between clinic staff or a current “Positive Peers” app user and an eligible patient. Although “Positive Peers” made use of geographically targeted and carefully targeted social media (ie, *Facebook*, *Instagram*, and *Twitter*) to support recruitment, these methods recruited few eligible users, primarily because of the restriction on where individuals received HIV care. Alternatively, clinic posters, clinician referrals, users’ word of mouth, and team attendance at local events, such as Cleveland’s Pride parade, produced the majority of enrolled participants. The rate of accrual was 6.4 new users a month over a 20-month enrollment period.

Potential participants contacted the app administrator directly to request eligibility confirmation via medical record review. Once eligibility was established, the administrator met with the potential participants to review the study evaluation procedures, secure informed consent, and review the app usage policies (see Multimedia Appendix 2). Posting policy clearly prohibits solicitation of sex or other inappropriate, discriminatory, hateful, or stigmatizing content on the *Community Forum*. All user communications are monitored by the administrator. To date, there have been no issues with inappropriate content.

**Figure 5:**
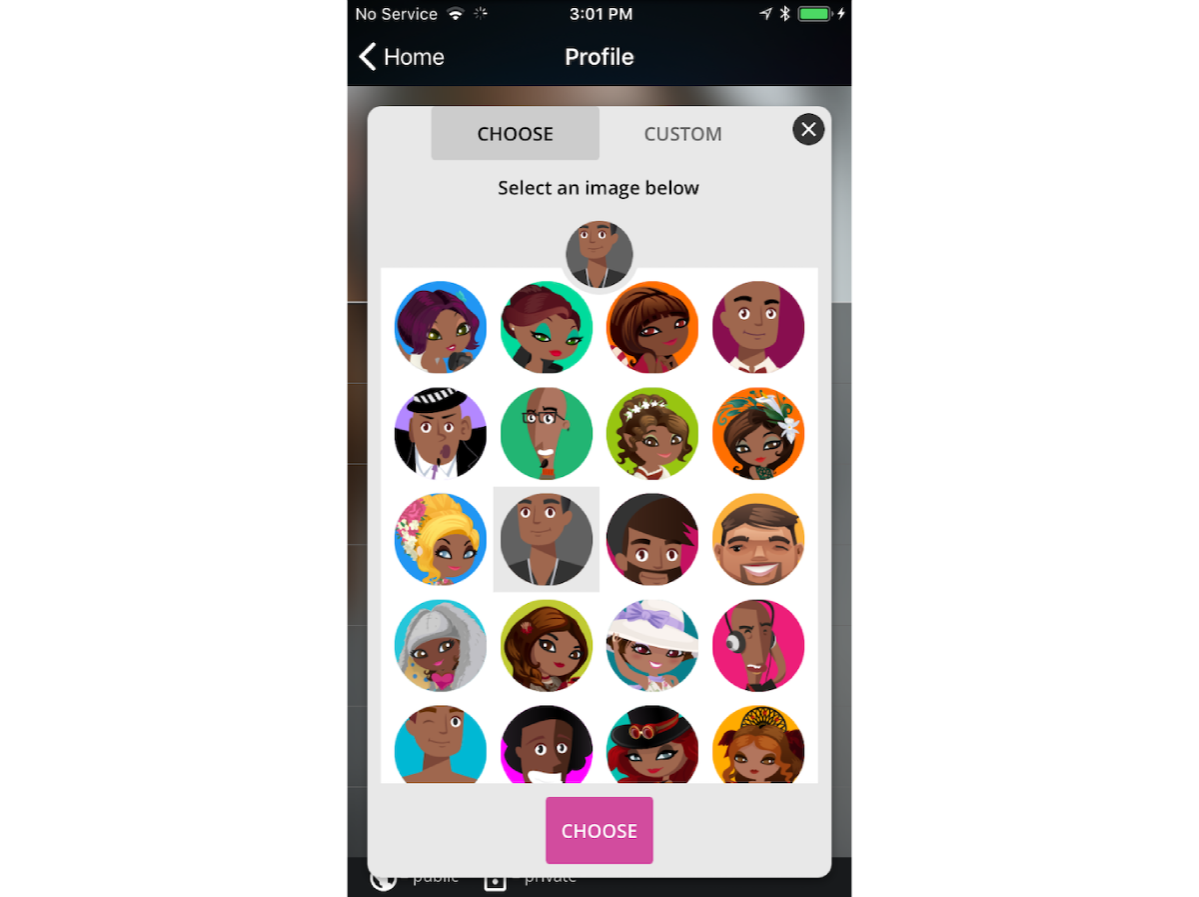
Screenshot of the “Positive Peers” avatar collection.

The “Positive Peers” app is designed to function on both Apple (iPhone Operating System [OS]) and Android platforms. Each enrolled user downloads the app on their own mobile device from the respective app provider for their OS, either Google Play or iTunes. A user’s virtual presence on the app is signified by a colorful contemporary cartoon visage that the user can choose from a large collection created by the design team (Figure 5). The style of these avatars, chosen by the CAB at the first meeting, reflects the demographic characteristics (eg, race and gender) and tastes (eg, fashion and attitude) of our young users.

The app administrator logs in at least daily for 5 days per week and monitors conversation posts on the community forum. If the participants feel that they are experiencing a mental health crisis, there are in-app directions to text 741741, a national suicide text line. A referral protocol was designed to aid the administrator in addressing or referring users’ concerns. All administrator communications are documented in a secure database and reviewed regularly at team meetings.

#### Retention

**Figure 6:**
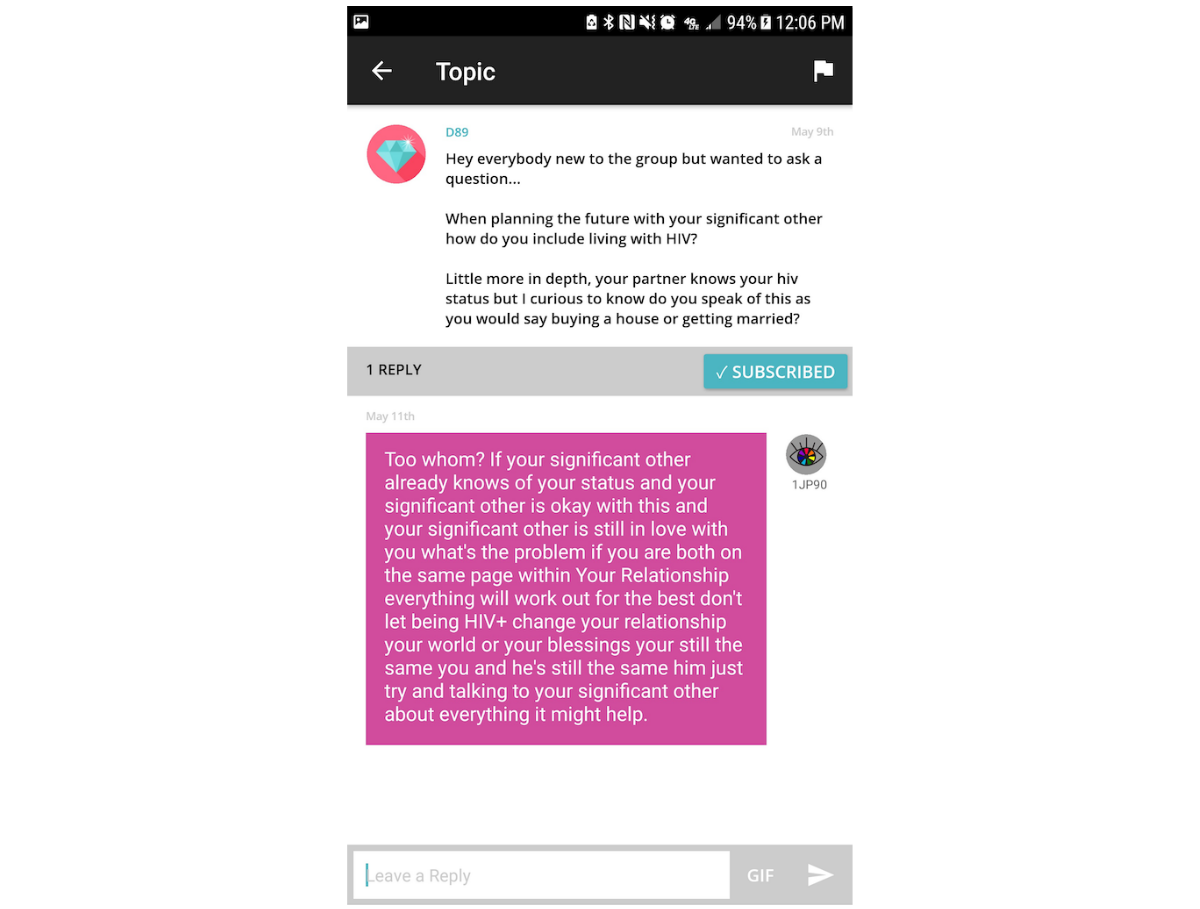
Screenshot of a community forum post from the admin.

The administrator engages in several activities to retain users and support app use. Following enrollment, the administrator remains available to the user by office phone, email, or chat to troubleshoot problems or reset forgotten passwords or usernames. In addition to sending *Daily Inspiration* push notifications, the administrator may start or respond to conversation threads on the community forum (eg, “Topic Tuesday;” Figure 6). The administrator maintains a visible presence in the user community and regularly attends clinic HIV support groups, community social and educational events for people living with HIV, and all “Positive Peers”–related functions. Importantly, the administrator makes an effort to check in with users not heard from over the previous month. This surveillance activity is intended to remind users of their involvement with the project and spur activity.

#### Compensation

Participants are compensated for the time they spend in each project evaluation activity, including prospective evaluation, qualitative interviews, and CAB activity. Participants were not compensated for using the app. Compensation is provided in the form of a US $25 gift card to either Target or Walmart. In addition, participant parking for meetings or data collection is validated, and local transportation vouchers are provided to commuters.

## Discussion

### Lessons Learned

Several important lessons have been revealed since “Positive Peers” began field testing in December 2016. These include cross-disciplinary challenges, hosting logistics associated with the medical center server, and participant barriers to use.

#### Cross-Disciplinary Collaboration

The members of our “Positive Peers” project team are professionally diverse and accustomed to different working styles and jargon. It was important to take time to learn these differences and adopt shared, team-defined methods for problem solving, tracking progress, and communicating needs. Inclusive meetings and messaging, detailed meeting notes from multiple participants, group decision making, Web-based electronic archives, and weekly contact help maintain an open, egalitarian group process that emphasizes nonsummative collaboration over siloed work. This approach is a significant resource toward the development of shared vision and consistent voice for “Positive Peers” activities, content, and evaluation.

#### Hosting Challenges

At implementation, all app metadata were hosted on a secure hospital server and tracked by *Google Analytics* (GA). After 5 months of weekly monitoring, metadata indicated that several user cases showed no log-in activity at all. This scenario triggered questioning as the user’s enrollment log-in should have been represented as user activity. In response, the digital design specialist and app administrator conducted tests with the archived, raw GA data and discovered that app log-ins that occurred on the hospital campus, or other free Wi-Fi services, were blocked from tracking by GA, our then primary source of user app activity data. Furthermore, GA tended to record additional session time as it did not *end* a session until the user logged out. GA counted app switching (eg, switch from “Positive Peers” to the phone to answer a call) and smartphone time-outs (without a log out) as time spent in “Positive Peers.” In response, a custom analytic system was created with the *Parse* open-source platform to collect app activity directly from the “Positive Peers” server.

#### User Barriers

App deletion by the user is a significant and unexpected barrier to using “Positive Peers.” Although users deleting the app to save device space is not unusual, the team was surprised at the regularity of this occurrence. This discovery was first observed during the early examination of the app metadata, which showed a large number of inactive cases. Upon exploration with the CAB and individual users, we discovered that our population not only deleted the app to make room for other apps but also lost, broke, and changed phones often; frequently forgot log-ins; and otherwise just forgot about the app. In response, the administrator monitored weekly activity and contacted those users to troubleshoot problems. Generally, the project team plans updates every 6 months to maintain interest, keep current with the OS changes, and address bugs.

Anonymity management was sometimes a barrier to app use for several users. Initially, the CAB requested the use of avatars to provide a layer of anonymity to all users. However, as the number of users grew, so did the demand to provide an option for uploading a profile picture and reveal additional demographic details in users’ profiles. Several users stated that they resisted in-app communications with each other as they were unsure of the identities of potential chat partners or community forum posters. Interpersonal communication theory suggests that this is a typical relational response [60,61]. Interpersonal relationship development is a behavioral process grounded in self-disclosure and incremental trust development. Social relationship development on social media can follow a similar pattern of intimate disclosure but may also include positive or entertaining disclosures as predictors of connection and closeness [62,63]. Although internet-based communication has been critiqued for disconnections between privacy values and disclosure behaviors, disclosure on social media has been shown to be a function of multiple privacy constructs, particularly privacy value and concern [64,65]. Given the ongoing stigma associated with HIV, we expected that our users would retain significant privacy concerns when communicating in-app. This challenge was addressed by organizing periodic in-person meetups, where our user community could learn more about each other. In addition, the app was changed to offer a choice to upload a selfie photo rather than cartoon avatar. Choosing when and how to share true identities affords our users additional control that they value.

Another challenge was jump-starting communication in the community forum. This is a primary app function, but at the outset, users were unsure who was using the app. Consequently, many users lurked instead of risking being the first to send a message. In an effort to increase interpersonal communication on the app, the administrator posted topics several times a week. Although this was successful at generating replies for a short period, the CAB ultimately provided feedback to stop the special posts as they were perceived as “the only posts (users) would ever see when logging in,” thus restricting the open dialog function of the community forum. The CAB further noted that there was a balance needed between the administrator-led conversations and the user-led conversations. After that, the administrator strived to do more replying than posting and limited posting to 1 to 3 posts a month.

### Next Steps

The immediate next step for “Positive Peers” dissemination and evaluation is the development of a virtual onboarding process that will allow “Positive Peers” to be available throughout the United States. This shift will reduce the responsibility of the app administrator (eg, user registration) but enable manageable growth with existing resources. Given the important cohesion that the app administrator generates, we are considering a tiered approach to administration that preserves a local contact but offloads some responsibilities to a central coordinator. Although bimonthly project team meetings continue, CAB meetings now occur on an ad hoc basis when a sufficiently developed agenda emerges from the ongoing activity. We expect that as the density of users increases, the CAB will be invigorated and will assume a more regular schedule. We will be testing long-distance conferencing technology to accomplish this aim.

At this writing, the final data collection for this first field experience with “Positive Peers” was near completion. Our evaluation of specific hypotheses and research questions are forthcoming, but given the burgeoning interests in locally designed and implemented health apps, we wished to provide others an overview of our development process and challenges.

### Conclusions

The “Positive Peers” mobile app aims to provide a secure virtual community tailored to those HIV subgroups most in need of attention and support. The development of “Positive Peers” was strongly collaborative, iterative, and time intensive. Continued development is bolstered by the interpersonal trust and cohesion created between the CAB and development team. The community of “Positive Peers” users made the request for this mobile app, informed its design, and continue to refine its function. Our cross-disciplinary approach provided specific expertise to create, implement, and evaluate this targeted technology.

## Appendix

Multimedia Appendix 1 “Positive Peers” demonstration video.

Multimedia Appendix 2 “Positive Peers” Dos and Don’ts for user posting.

## Abbreviations

CAB: community advisory board
CEO: chief executive officer
GA: Google Analytics
HAB: HIV/AIDS Bureau
HRSA: Health Resources and Services Administration
LGBTQ: Lesbian, Gay, Bisexual, Trans, or Queer
OS: operating system
SPNS: Special Projects of National Significance
YPLWH: young people living with HIV

## References

[1] (2019). Centers for Disease Control and Prevention (CDC).

[2] Centers for Disease Control and Prevention (CDC).

[3] Beer L, Mattson CL, Shouse RL, Prejean J (2016). Receipt of clinical and prevention services, clinical outcomes, and sexual risk behaviors among HIV-infected young adults in care in the United States. AIDS Care.

[4] Ayres JR, Paiva V, França I, Gravato N, Lacerda R, Negra MD, Marques HH, Galano E, Lecussan P, Segurado AC, Silva MH (2006). Vulnerability, human rights, and comprehensive health care needs of young people living with HIV/AIDS. Am J Public Health.

[5] Zanoni BC, Mayer KH (2014). The adolescent and young adult HIV cascade of care in the United States: exaggerated health disparities. AIDS Patient Care STDS.

[6] (2018). Centers for Disease Control and Prevention.

[7] Boyer CB, Greenberg L, Chutuape K, Walker B, Monte D, Kirk J, Ellen JM, Adolescent Medicine Trials Network (2017). Exchange of sex for drugs or money in adolescents and young adults: an examination of sociodemographic factors, HIV-related risk, and community context. J Community Health.

[8] Buchacz K, Armon C, Tedaldi E, Palella FJ, Novak RM, Ward D, Hart R, Durham MD, Brooks JT (2018). Disparities in HIV viral load suppression by race/ethnicity among men who have sex with men in the HIV outpatient study. AIDS Res Hum Retroviruses.

[9] Iroh PA, Mayo H, Nijhawan AE (2015). The HIV care cascade before, during, and after incarceration: a systematic review and data synthesis. Am J Public Health.

[10] Crepaz N, Dong X, Wang X, Hernandez AL, Hall HI (2018). Racial and ethnic disparities in sustained viral suppression and transmission risk potential among persons receiving HIV care - United States, 2014. MMWR Morb Mortal Wkly Rep.

[11] Vanable PA, Carey MP, Blair DC, Littlewood RA (2006). Impact of HIV-related stigma on health behaviors and psychological adjustment among HIV-positive men and women. AIDS Behav.

[12] Yehia BR, Rebeiro P, Althoff KN, Agwu AL, Horberg MA, Samji H, Napravnik S, Mayer K, Tedaldi E, Silverberg MJ, Thorne JE, Burchell AN, Rourke SB, Rachlis A, Mayor A, Gill MJ, Zinski A, Ohl M, Anastos K, Abraham AG, Kitahata MM, Moore RD, Gebo KA, North American AIDS Cohort Collaboration on Research and Design (NA-ACCORD) (2015). Impact of age on retention in care and viral suppression. J Acquir Immune Defic Syndr.

[13] Martinez J, Chakraborty R, American Academy of Pediatrics Committee on Pediatric Aids (2014). Psychosocial support for youth living with HIV. Pediatrics.

[14] Rebeiro PF, McPherson TD, Goggins KM, Turner M, Bebawy SS, Rogers WB, Brinkley-Rubinstein L, Person AK, Sterling TR, Kripalani S, Pettit AC (2018). Health literacy and demographic disparities in HIV care continuum outcomes. AIDS Behav.

[15] Kaiser Family Foundation (2017). Kaiser Family Foundation.

[16] Li H, Li X, Zhang L, Chow E (2016). Effects of multiple types of stigma on the probability of HIV disclosure to sex partners: a systematic review. Sex Health.

[17] Chaudoir SR, Fisher JD, Simoni JM (2011). Understanding HIV disclosure: a review and application of the Disclosure Processes Model. Soc Sci Med.

[18] Enane LA, Vreeman RC, Foster C (2018). Retention and adherence: global challenges for the long-term care of adolescents and young adults living with HIV. Curr Opin HIV AIDS.

[19] Lall P, Lim SH, Khairuddin N, Kamarulzaman A (2015). Review: an urgent need for research on factors impacting adherence to and retention in care among HIV-positive youth and adolescents from key populations. J Int AIDS Soc.

[20] Hull MW, Wu Z, Montaner JS (2012). Optimizing the engagement of care cascade: a critical step to maximize the impact of HIV treatment as prevention. Curr Opin HIV AIDS.

[21] Ryscavage PA, Anderson EJ, Sutton SH, Reddy S, Taiwo B (2011). Clinical outcomes of adolescents and young adults in adult HIV care. J Acquir Immune Defic Syndr.

[22] Muthulingam D, Chin J, Hsu L, Scheer S, Schwarcz S (2013). Disparities in engagement in care and viral suppression among persons with HIV. J Acquir Immune Defic Syndr.

[23] Gardner EM, McLees MP, Steiner JF, Del Rio C, Burman WJ (2011). The spectrum of engagement in HIV care and its relevance to test-and-treat strategies for prevention of HIV infection. Clin Infect Dis.

[24] Bangsberg DR, Ragland K, Monk A, Deeks SG (2010). A single tablet regimen is associated with higher adherence and viral suppression than multiple tablet regimens in HIV+ homeless and marginally housed people. AIDS.

[25] Labhardt ND, Ringera I, Lejone TI, Klimkait T, Muhairwe J, Amstutz A, Glass TR (2018). Effect of offering same-day ART vs usual health facility referral during home-based HIV testing on linkage to care and viral suppression among adults with HIV in Lesotho: The CASCADE Randomized Clinical Trial. J Am Med Assoc.

[26] McNulty MC, Schneider JA (2018). Care continuum entry interventions: seek and test strategies to engage persons most impacted by HIV within the United States. AIDS.

[27] Catalani C, Philbrick W, Fraser H, Mechael P, Israelski DM (2013). mHealth for HIV treatment & prevention: a systematic review of the literature. Open AIDS J.

[28] Dillingham R, Ingersoll K, Flickinger TE, Waldman AL, Grabowski M, Laurence C, Wispelwey E, Reynolds G, Conaway M, Cohn WF (2018). PositiveLinks: a mobile health intervention for retention in HIV care and clinical outcomes with 12-month follow-up. AIDS Patient Care STDS.

[29] Whitehead L, Seaton P (2016). The effectiveness of self-management mobile phone and tablet apps in long-term condition management: a systematic review. J Med Internet Res.

[30] Navarra AD, Gwadz MV, Whittemore R, Bakken SR, Cleland CM, Burleson W, Jacobs SK, Melkus GD (2017). Health technology-enabled interventions for adherence support and retention in care among US HIV-infected adolescents and young adults: an integrative review. AIDS Behav.

[31] LeGrand S, Muessig KE, Platt A, Soni K, Egger JR, Nwoko N, McNulty T, Hightow-Weidman LB (2018). Epic allies, a gamified mobile phone app to improve engagement in care, antiretroviral uptake, and adherence among young men who have sex with men and young transgender women who have sex with men: protocol for a randomized controlled trial. JMIR Res Protoc.

[32] Flickinger TE, DeBolt C, Waldman AL, Reynolds G, Cohn WF, Beach MC, Ingersoll K, Dillingham R (2017). Social support in a virtual community: analysis of a clinic-affiliated online support group for persons living with HIV/AIDS. AIDS Behav.

[33] Holt-Lunstad J, Robles TF, Sbarra DA (2017). Advancing social connection as a public health priority in the United States. Am Psychol.

[34] Bekele T, Rourke SB, Tucker R, Greene S, Sobota M, Koornstra J, Monette L, Rueda S, Bacon J, Watson J, Hwang SW, Dunn J, Guenter D, Positive Spaces Healthy Places Team (2013). Direct and indirect effects of perceived social support on health-related quality of life in persons living with HIV/AIDS. AIDS Care.

[35] Ozbay F, Fitterling H, Charney D, Southwick S (2008). Social support and resilience to stress across the life span: a neurobiologic framework. Curr Psychiatry Rep.

[36] Taylor SE, Friedman HS (2011). Social support: a review. The Oxford Handbook of Health Psychology.

[37] Ybarra ML, Prescott TL, Philips GL, Bull SS, Parsons JT, Mustanski B (2016). Iteratively developing an mHealth HIV prevention program for sexual minority adolescent men. AIDS Behav.

[38] Goldenberg T, McDougal SJ, Sullivan PS, Stekler JD, Stephenson R (2015). Building a mobile HIV prevention app for men who have sex with men: an iterative and community-driven process. JMIR Public Health Surveill.

[39] Kumar S, Nilsen WJ, Abernethy A, Atienza A, Patrick K, Pavel M, Riley WT, Shar A, Spring B, Spruijt-Metz D, Hedeker D, Honavar V, Kravitz R, Lefebvre RC, Mohr DC, Murphy SA, Quinn C, Shusterman V, Swendeman D (2013). Mobile health technology evaluation: the mHealth evidence workshop. Am J Prev Med.

[40] Muessig KE, LeGrand S, Horvath KJ, Bauermeister JA, Hightow-Weidman LB (2017). Recent mobile health interventions to support medication adherence among HIV-positive MSM. Curr Opin HIV AIDS.

[41] Shaw S, Amico KR (2016). Antiretroviral therapy adherence enhancing interventions for adolescents and young adults 13-24 years of age: a review of the evidence base. J Acquir Immune Defic Syndr.

[42] Erguera XA, Johnson MO, Neilands TB, Ruel T, Berrean B, Thomas S, Saberi P (2019). WYZ: a pilot study protocol for designing and developing a mobile health application for engagement in HIV care and medication adherence in youth and young adults living with HIV. BMJ Open.

[43] Guilamo-Ramos V, Thimm-Kaiser M, Benzekri A, Futterman D (2019). National Academy of Medicine.

[44] Gardner M, Steinberg L (2005). Peer influence on risk taking, risk preference, and risky decision making in adolescence and adulthood: an experimental study. Dev Psychol.

[45] Sherman LE, Payton AA, Hernandez LM, Greenfield PM, Dapretto M (2016). The power of the like in adolescence: effects of peer influence on neural and behavioral responses to social media. Psychol Sci.

[46] Yu AY, Tian SW, Vogel D, Kwok R (2010). Can learning be virtually boosted? An investigation of online social networking impacts. Comput Educ.

[47] DeAndrea DC, Ellison NB, LaRose R, Steinfield C, Fiore A (2012). Serious social media: on the use of social media for improving students' adjustment to college. Internet High Educ.

[48] Fox J, McEwan B (2017). Distinguishing technologies for social interaction: the perceived social affordances of communication channels scale. Commun Monogr.

[49] Evans SK, Pearce KE, Vitak J, Treem JW (2017). Explicating affordances: a conceptual framework for understanding affordances in communication research. J Comput-Mediat Commun.

[50] Rice RE, Evans SK, Pearce KE, Sivunen A, Vitak J, Treem JW (2017). Organizational media affordances: operationalization and associations with media use. J Commun.

[51] Sundar SS, Metzger MJ, Flanagin A, Eastin MS, Rieh SY, Hilligoss B (2008). The MAIN model: A heuristic approach to understanding technology effects on credibility. Digital Media,Youth, and Credibility (The John D. and Catherine T. MacArthur Foundation Series on Digital Media and Learning).

[52] Schrock Ar (2015). Communicative affordances of mobile media: portability, availability, locatability, and multimediality. Int J Commun.

[53] McCurdie T, Taneva S, Casselman M, Yeung M, McDaniel C, Ho W, Cafazzo J (2012). mHealth consumer apps: the case for user-centered design. Biomed Instrum Technol.

[54] Abras C, Maloney-Krichmar D, Preece J (2004). Encyclopedia of Human-Computer Interaction.

[55] de Vito Dabbs A, Myers BA, Mc Curry KR, Dunbar-Jacob J, Hawkins RP, Begey A, Dew MA (2009). User-centered design and interactive health technologies for patients. Comput Inform Nurs.

[56] Free C, Phillips G, Watson L, Galli L, Felix L, Edwards P, Patel V, Haines A (2013). The effectiveness of mobile-health technologies to improve health care service delivery processes: a systematic review and meta-analysis. PLoS Med.

[57] Avery A, McMillen Smith J, Kratz J, Briggs J, Step M Positive Peers.

[58] Walsh TM, Volsko TA (2008). Readability assessment of internet-based consumer health information. Respir Care.

[59] Webel AR, Wantland D, Rose CD, Kemppainen J, Holzemer WL, Chen W, Johnson MO, Nicholas P, Eller LS, Chaiphibalsarisdi P, Sefcik E, Nokes K, Corless IB, Tyer-Viola L, Kirksey K, Voss J, Sullivan K, Rivero-Méndez M, Brion J, Iipinge S, Phillips JC, Portillo C (2015). A cross-sectional relationship between social capital, self-compassion, and perceived HIV symptoms. J Pain Symptom Manage.

[60] Altman I, Taylor D (1973). Social Penetration: The Development of Interpersonal Relationships.

[61] Sprecher S, Treger S, Wondra JD, Hilaire N, Wallpe K (2013). Taking turns: reciprocal self-disclosure promotes liking in initial interactions. J Exp Soc Psychol.

[62] Dindia K, Allen M, Preiss R, Gayle B, Burrell N, Allen M, Preiss RW, Gayle BM, Burrell N (2002). Self-disclosure research: knowledge through meta-analysis. Interpersonal Communication Research: Advances Through Meta-analysis.

[63] Utz S (2015). The function of self-disclosure on social network sites: not only intimate, but also positive and entertaining self-disclosures increase the feeling of connection. Comput Hum Behav.

[64] Nemec Zlatolas L, Welzer T, Heričko M, Hölbl M (2015). Privacy antecedents for SNS self-disclosure: the case of Facebook. Comput Hum Behav.

[65] Gross R, Acquisti A (2005). Information Revelation and Privacy in Online Social Networks. Proceedings of the 2005 ACM workshop on Privacy in the electronic society.

