# Supplementary material for: “Positive Peers”: Function and Content Development of a Mobile App for Engaging and Retaining Young Adults in HIV Care
Source: JMIR Form Res. 2020 Jan 30;4(1):e13495. doi: 10.2196/13495 (PMC7055772; doi:10.2196/13495)
Supplement: Multimedia Appendix 2 [file formative_v4i1e13495_app2.docx]

**Appendix C**


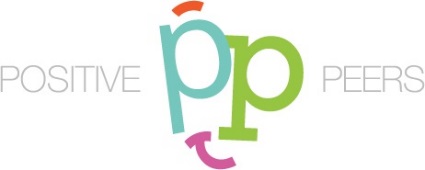
**The Do’s & Don’ts**

***How To Make The Most Of The App***

**Do’s**

To ensure the best experience for you and others here are a few things we encourage you to do and do often:

1. **Log in every day**

Logging in every day earns you and the community more *health score* points as well and keeps you fresh with all the latest news, funny posts, and inspiring stories. Plus, your new friends will appreciate seeing your avatar’s beautiful smiling face on a daily basis ☺

**2. Keep track**

Positive Peers has several built -in tools to help you quickly and efficiently manage your health. Keeping track of your exercise, medication adherence, meetings / appointments, and other support and social events through positive Peers is easy and fun. It just so happens to score you some more health points too, giving you a little some-some to share in *my community* as encouragement to others! You’re just awesome, what can we say.

**3. Go post crazy!**

Have an important question you’ve been itching to ask? An inspiring story you’ve been dying to share? Or how about a burning desire to geek-out over the latest episode of your favorite TV show? These are all great reasons to post in *my community*. Whether you’re on a quest for knowledge or just want a chance to chit-chat with others about your love of Beyonce, the *my community* is the place to go to scratch that itch. Who knows, perhaps you’ll make a new friend to chat and catch a movie with!

**4. Get inspired**

Positive Peers’ *resource* page can connect you to an ever growing world of inspiring, funny, and informative videos and blogs. This perfect blend of hilarious yet accurate content will keep you well informed about topics such as living with HIV, relationships and intimacy, and good nutrition and exercise. It’s also home to Tales of Triumph, a collection of personal success stories by members of the Positive Peers community. Read up, check back often for boat loads of new content, and get inspired!

**Don’ts**

Positive Peers is a safe and supportive community where people encourage, inspire, and respect one another. It’s a place for shared laughter and shared humanity. As such, here are a few things you should avoid:

**1. Bullying**

Bullying will not be tolerated, period. We are a community in which there is a great respect for the dignity, privacy, and humanity of every individual. Please avoid intimidating, or using hurtful and offensive language.

**2. Harassment / Stalking**

Nobody likes being harassed. Part of what makes our community such a comfortable and welcoming place is the warm, friendly, and respectful attitudes of fellow community members. As such, please avoid any aggressive behavior, intimidation, and/or unwanted advances.

**3. Partner Seeking**

The Positive Peers app is not the right place to actively seek a new date, sex partner, or significant other. While we encourage you to be compassionate, friendly, and social, there will be no toleration of partner seeking within Positive Peers community.

**Note:** [Name], the application administrator, will be monitoring use of the Positive Peers app, engaging with users, and replying to inquiries/issues from the hours of 8am – 4:30pm Monday through Friday.

* Exceptions include vacation days and holidays *(Christmas, Thanksgiving, Labor Day, Independence Day, Memorial Day, and New Years Day)*

**Note:** Any violation of the above will result in discipline, including but not limited to removal from the Positive Peers community. Should you witness or being made uncomfortable by any of the above, please contact the app administrator, [Name], within the app itself, at [positivepeers@metrohealth.org](mailto:positivepeers@metrohealth.org), or at 216-778-8137.

**
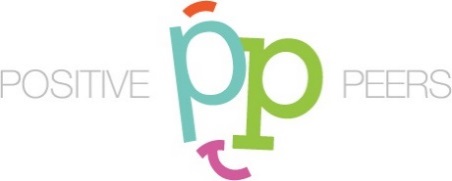
Acknowledgement**

*Of User Guide Do’s & Don’ts*

**System of Enforcement**

Participants will be withdrawn if they are unable to follow the stated guidelines for application participation (e.g., bullying, partner seeking activity). In the event of an offense, the app administrator will warn users through a tiered violation system:

**First offense:** Warning

**Second offense:** The user is locked out of the app until they have an in-person or phone discussion with Josh Kratz

**Third offense:** The user is removed from the app and the study

**Agreement to Positive Peers User Guide**

I have read, understand, and agree to the Positive Peers User Guide.

Participant Name: _________________________________

Participant Signature: ______________________________ Date: _____________
